# Pediatric severe TBI in South America: Healthcare resource utilization before and during the COVID-19 pandemic

**DOI:** 10.1371/journal.pgph.0004318

**Published:** 2025-05-08

**Authors:** Janessa M. Graves, Silvia Lujan, Julia L. Velonjara, Gustavo Petroni, Nahuel Guadagnoli, Michael J. Bell, PEGASUS Argentina author group, Monica S. Vavilala

**Affiliations:** 1 WWAMI Rural Health Research Center, Department of Family Medicine, School of Medicine, University of Washington, Seattle, Washington, United States of America; 2 Washington State University College of Nursing, Spokane, Washington, United States of America; 3 Harborview Injury Prevention and Research Center, University of Washington, Seattle, Washington, United States of America; 4 Centro de Informática e Investigación Clínica, Rosario, Santa Fe, Argentina; 5 Department of Anesthesiology & Pain Medicine, School of Medicine, University of Washington, Seattle, Washington, United States of America; 6 Children’s National Hospital Critical Care Medicine, Washington, District of Columbia, United States of America; Royal Infirmary of Edinburgh, UNITED KINGDOM OF GREAT BRITAIN AND NORTHERN IRELAND

## Abstract

Traumatic brain injury (TBI) is a leading cause of pediatric morbidity and mortality worldwide. Understanding healthcare utilization during hospitalization for severe TBI across varied resource settings is crucial for informing improvements in clinical practice, patient outcomes, and for reducing TBI burden. We examined hospital services utilization among children with severe TBI in South America. This secondary analysis of data collected during the baseline period of a randomized controlled trial implementing severe TBI clinical management guidelines identified pediatric patients (<18 years) with severe TBI across 16 hospitals in Argentina, Chile, and Paraguay between September 1, 2019 and July 13, 2020. Demographics, injury characteristics, clinical presentation, intensive care unit (ICU) utilization, surgical interventions, and imaging data were collected, and descriptive statistics were calculated. Utilization differences were examined across two time periods based on the COVID-19 pandemic: prepandemic (September 1, 2019-March 10, 2020) and during the pandemic (March 11-July 13, 2020) using Student’s t-tests and chi-square tests. One-hundred and sixteen patients (median age: 6.5 years) with severe TBI enrolled during the study (n = 80 prepandemic and n = 36 during the pandemic) period. There were no differences in demographic characteristics, injury mechanism, or discharge outcomes between time periods. Vasopressor use was less common during the pandemic (63.8% vs. 33.3%, p < 0.01) as were surgeries (p = 0.05). Other measures, including nutrition, mechanical ventilation, and central venous pressure monitoring were stable over time. Intracranial pressure (ICP) monitoring remained above 50% throughout. We failed to detect a pandemic effect resulting in pediatric severe TBI ICU care or hospital discharge outcome changes. Clinicians adapted successfully.

## Introduction

Traumatic brain injury (TBI) is a leading cause of pediatric morbidity and mortality worldwide [1]. Each year, over three million children sustain a TBI, and the incidence of TBI around the world varies widely between countries and regions [1,2]. Common causes of TBI include falls, motor vehicle crashes, abuse, and sports-related injuries [1,2]. In South America, motor vehicle crashes and falls comprise a majority of TBI [3,4].

Severe TBI, characterized by serious acute symptoms, extended unconsciousness or severely altered consciousness, comprises only a fraction of TBIs [5]; however, they are associated with high rates of morbidity and mortality, especially in low- and middle-income countries [6,7]. For example, in Argentina, which is classified as upper-middle-income, where the estimated incidence of TBI was 228 per 100,000 (2016 estimate; 95% CI: 192–273) [2], hospital care for severe TBI patients can be hindered by significant challenges, including inadequate staffing, resources, and availability of services [8]. Recent efforts to understand resource limitations in South American countries, including Argentina, provide insight into the challenges in providing optimal, guideline-adherent care to severe TBI patients [7–9].

Understanding the utilization of healthcare services during hospitalization for severe TBI is crucial for informing improvements in clinical practice, improving patient outcomes, and for reducing the burden of TBI. Established guidelines, such as those published by the Brain Trauma Foundation (BTF), provide evidence-based recommendations for the ICU care management of pediatric severe TBI [10]. However, these guidelines (and others) have been developed in the context of developed healthcare systems with established infrastructure and capabilities that are not available in resource-limited settings [7].

Few studies have described the hospital services utilization among children with severe TBI in the South American context. De Almeida and colleagues provided epidemiological and economic data on pediatric severe TBI in Brazil; however, their analysis did not provide granular information regarding in-hospital resource utilization [11]. One study of the in-hospital management of 34 Colombian children with severe TBI from 2010-2015 reported that intracranial pressure (ICP) monitoring was used in 14% of cases [12]. Surgical ICP management, such as decompressive craniectomy, was also infrequently used (9%) in favor of pharmacological management (e.g., hypertonic saline and/or mannitol) (94%) [12]. Previous research from Argentina provides similar additional data on hospital services utilization for pediatric severe TBI in 2011–2013. In that study, decompressive craniectomy was conducted in 12% of patients; however, ICP monitoring was reported in 60% of cases [13].

The COVID-19 pandemic likely exacerbated existing challenges in treating pediatric trauma patients in low-resource settings. However, the literature on healthcare resource utilization for pediatric trauma or TBI during the pandemic remains limited. One qualitative study found that Mozambican hospital staff reported equipment and medication shortages hindered care for children with traumatic injuries, [14] though it neither specifically addressed TBI nor focused on the context of pediatric trauma care in South American countries, primarily Argentina. Evidence exists, however, to suggest that the pandemic contributed to treatment disruptions for pediatric oncology patients in South American countries [15]. The purpose of this retrospective cohort study was to provide updated data on healthcare resource utilization among hospitalized children with severe TBI in South America before and during the COVID-19 pandemic.

## Methods

This study utilized secondary data collected during the baseline period of a randomized controlled trial (RCT) in South America (ClinicalTrials.gov ID NCT03896789). The Pediatric Guideline Adherence and Outcomes (PEGASUS) program is a severe TBI management program based on implementation science principles and evidence-based severe TBI clinical management guidelines, like BTF guidelines. The PEGASUS program was initially designed and pilot-tested in the United States and was associated with adherence to key performance indicators and improvements in patient outcomes at no increased cost [16,17]. In 2019, to test PEGASUS intervention effectiveness more broadly, a multi-site RCT was launched in Argentina, Chile, and Paraguay [18]. During the initial RCT baseline period, prior to program implementation, hospital utilization data were collected for a 10.5-month period, from September 1, 2019 to July 13, 2020. This retrospective cohort study relies on baseline PEGASUS trial data.

### Study participants

Participants were screened and enrolled in the PEGASUS study at time of admission to a study hospital pediatric intensive care unit (PICU) with severe TBI, defined as a CT scan indicative of TBI and a GCS ≤ 8 at any point during or after admission, and aged < 18 years. All eligible patients and their guardians were approached for consent and patients were included unless they declined to participate or died before consent was obtained. There were no other exclusions.

### Measures

The PEGASUS study team collected data upon admission for all pediatric severe TBI patients presenting at one of the 16 study site hospitals located in Chile, Paraguay, and throughout nine provinces in north and central Argentina. Study site hospitals, comprised of ten pediatric only and six mixed adult and pediatric facilities, all have pediatric intensive care units and regularly treat pediatric patients with severe TBI.

The following data were collected: demographic characteristics, injury characteristics, clinical presentation, outcomes, hospital and PICU utilization, surgical interventions, computed tomography (CT) use, and various other measures to evaluate health services utilization, such as days on a ventilator. Count utilization variables (such as surgical interventions or CT imaging) are reported as the total number performed each PICU stay. Abbreviated injury scores (AIS) were calculated for each injured body region and an overall Injury Severity Score (ISS) calculated for each patient [19]. Data were collected and stored using standardized study-specific data entry forms in a secure, online clinical data management platform.

### Analysis

Study measures were summarized using descriptive statistics, including frequencies for categorical variables and medians, interquartile ranges, and ranges for continuous variables. We reported results across all patients for the study period from September 1, 2019, to July 13, 2020. To examine potential impacts of the COVID-19 pandemic on utilization, we stratified patients enrolled prior to the World Health Organization declaration of the pandemic on March 11, 2020 (“prepandemic” period) and afterwards (“during pandemic” period). Differences in measures were compared across time periods using chi-square tests (or Fisher’s Exact tests when applicable) for categorical variables and Student’s t-tests for continuous variables. All analyses were conducted in SAS Software Version 9.4 (SAS Institute Inc., Cary, NC, USA). Study procedures were reviewed and approved as minimal risk by the institutional review board at the [institution blinded for peer review] and the 16 study hospitals for local recruitment, consent, and activities (Clinicaltrails.gov identifier: blinded for peer review).

## Results

A total of 116 pediatric patients with severe TBI were admitted to participating hospitals between September 1, 2019, and July 13, 2020. Two-thirds (n = 80) were admitted prior to the WHO COVID-19 pandemic declaration on March 11, 2020; 31.0% (n = 36) were admitted during the pandemic.

Patients were predominantly male and had a median of 6.5 years (Table 1). Public ways and homes were the most common location of injury, with traffic incidents, fall from height, and struck with an object being the most common injury causes. Overall, 71.6% (n = 83) patients had a severe GCS score upon admission. Nearly all patients (82.8%) were discharged home following their hospital stay. Patients admitted prior to and during the pandemic did not significantly differ in terms of demographics, injury characteristics, clinical presentation, or disposition (Table 1).

**Table 1:** Characteristics of pediatric patients with severe traumatic brain injury in South America, September 1, 2019-July 13, 2020 (N = 116).

	Prepandemic (9/1/19–3/10/20) (n = 80)	During pandemic (3/11/20-7/13/20) (n = 36)	Total (9/1/19–7/13/20) (N = 116)	
	Count (%)	Count (%)	Count (%)	Sig.
**Demographic and injury information**				
Age (years)				
Median (IQR)	5.0 (8.5)	8.0 (12.0)	6.5 (11.0)	
Range	0.2-17.0	0.2-16.0	0.2-17.0	
Sex				0.40
Male	49 (61.3)	25 (69.4)	74 (63.8)	
Female	31 (38.7)	11 (30.6)	42 (36.2)	
Place (location) of injury				0.54
Public way	43 (53.8)	14 (38.9)	57 (49.1)	
Public place	4 (5.0)	2 (5.6)	6 (5.2)	
Home	30 (37.5)	19 (52.8)	49 (42.2)	
School	1 (1.3)	0 (0.0)	1 (0.9)	
Sports/recreation	1 (1.3)	1 (2.8)	2 (1.7)	
Other	1 (1.3)	0 (0.0)	1 (0.9)	
Cause of injury				0.36
Traffic accident	37 (46.3)	13 (36.1)	50 (43.1)	
Fall from height	21 (26.3)	10 (27.8)	31 (26.7)	
Own fall height	1 (1.3)	2 (5.6)	3 (2.6)	
Struck with an object	9 (11.3)	7 (19.4)	16 (13.8)	
Suicide	0 (0.0)	1 (2.8)	1 (0.9)	
Gunshot wound	5 (6.3)	1 (2.8)	6 (5.2)	
Other	7 (8.8)	2 (5.6)	9 (7.8)	
Transfer from other hospital				0.35
No	20 (25.0)	12 (33.3)	32 (27.6)	
Yes	60 (75.0)	24 (66.7)	84 (72.4)	
Transportation to hospital^1^				0.78
Ambulance, with physician	29 (36.3)	11 (30.6)	40 (34.5)	
Ambulance, no physician	3 (3.8)	2 (5.6)	5 (4.3)	
Firemen	1 (1.3)	2 (5.6)	3 (2.6)	
Car	29 (36.3)	16 (44.4)	45 (38.8)	
By foot	3 (3.8)	1 (2.8)	4 (3.5)	
Police	1 (1.3)	0 (0.0)	1 (0.9)	
Unknown	11 (13.8)	4 (11.1)	15 (12.9)	
Other	3 (3.8)	0 (0.0)	3 (2.6)	
Injury to first admission time (hours)				
Median (IQR)	1.3 (3.0)	1.1 (1.6)	1.2 (2.8)	
Range	0.2-13.4	0.0-18.0	0.0-18.0	
**Clinical presentation**				
Glasgow Coma Scale (GCS) Score				0.14
Mild (13–15)	12 (15.0)	1 (2.8)	13 (11.2)	
Moderate (9–12)	14 (17.5)	6 (16.7)	20 (17.2)	
Severe (3–8)	54 (67.5)	29 (80.6)	83 (71.6)	
Injury Severity Score (ISS)				
Median (IQR)	21.0 (18.0)	25.0 (15.0)	25.0 (17.5)	
Range	1.0-75.0	3.0-75.0	1.0-75.0	
Max non-head abbreviated injury score (AIS)				0.77
None (0)	24 (30.0)	14 (38.9)	38 (32.8)	
Minor (1)	14 (17.5)	6 (16.7)	20 (17.2)	
Moderate (2)	13 (16.3)	6 (16.7)	19 (16.4)	
Serious (3)	11 (13.8)	2 (5.6)	13 (11.2)	
Severe (4)	9 (11.3)	6 (16.7)	15 (12.9)	
Critical (5)	6 (7.5)	1 (2.8)	7 (6.0)	
Fatal (6)	3 (3.8)	1 (2.8)	4 (3.5)	
**Injury outcomes**				
Discharge disposition				0.26
Home without homecare	27 (33.8)	18 (50.0)	45 (38.8)	
Home with homecare	36 (45.0)	15 (41.7)	51 (44.0)	
Other hospital	7 (8.7)	0 (0.0)	7 (6.0)	
Other	3 (3.8)	1 (2.8)	4 (3.5)	
Not applicable^2^	7 (8.7)	2 (5.6)	9 (7.8)	

Unless otherwise indicated, values represent counts (n) and percentages. Within-category column totals may not sum to 100% due to missing data or rounding. Significance (sig.) represents p-value from chi-squared tests for categorical measures.

^1^Transportation to first hospital visited (71.7% of all cases involved transfer from first hospital visited to trauma center).

^2^Patients who died during admission.

Healthcare service utilization following admission for severe TBI is reported in Table 2. Within the PICU, nutrition was commonly provided, usually delivered via enteral feeding (Table 2). Approximately half of patients received vasopressor therapy overall, but this varied by time period. Prior to the pandemic, 63.8% of patients received vasopressor therapy, compared to 33.3% during the pandemic (p = 0.002). Surgical procedures also differed by time period, with significantly more patients not receiving surgery during the pandemic than before (41.7% vs. 18.8%, respectively; p = 0.047). Invasive arterial cannulation was more common prepandemic compared to during the pandemic period, but this difference was not statistically significant. Patients stayed in the hospital for a median 20.5 days (mean: 22.2); the longest length of stay was 123.3 days. Median PICU length of stay was 184.5 hours (mean: 294.0). Neither the hospital nor the PICU lengths of stay differed between the prepandemic and pandemic time periods. There was no difference in other healthcare utilization parameters examined.

**Table 2:** Healthcare service utilization of pediatric patients with severe traumatic brain injury in South America, September 1, 2019-July 13, 2020 (N = 116).

	Prepandemic (9/1/19–3/10/20) (n = 80)	During pandemic (3/11/20-7/13/20) (n = 36)	Total (9/1/19–7/13/20) (N = 116)	Sig.
	**Count (%)**	**Count (%)**	**Count (%)**	
**Hospital Utilization**				
Hospital LOS (days)				
Median (IQR)	15.9 (20.8)	13.7 (21.5)	15.7 (20.9)	
Range	0.6-123.3	1.2-75.3	0.6-123.3	
**ICU Utilization**				
ICU LOS (hours)				
Median (IQR)	158.0 (270.5)	197.5 (322.5)	184.5 (292.0)	
Range	13.0-1817.0	28.0-1199.0	13.0-1817.0	
Nutrition				1.00
No	8 (10.0)	4 (11.1)	12 (10.3)	
Yes	72 (90.0)	32 (88.9)	104 (89.7)	
Parenteral feeding				0.16
No	73 (92.4)	36 (100.0)	109 (94.8)	
Yes	6 (7.6)	0 (0.0)	6 (5.2)	
Enteral feeding				0.70
No	13 (17.4)	7 (19.4)	20 (17.4)	
Yes	66 (83.5)	29 (80.6)	95 (82.6)	
Vasopressor days				<0.01
Any (>0)	51 (63.8)	12 (33.3)	63 (54.3)	
None	29 (36.3)	24 (66.7)	53 (45.7)	
Hypersomolar days				0.62
Any (>0)	19 (23.8)	7 (19.4)	26 (22.4)	
None	61 (76.3)	29 (80.6)	90 (77.6)	
Barbiturates used in ICU (days)				0.54
Any (>0)	11 (13.8)	3 (8.3)	14 (12.1)	
None	69 (86.3)	33 (91.7)	102 (87.9)	
**Surgical**				
Number of surgical procedures				0.05
0	15 (18.8)	15 (41.7)	30 (25.9)	
1	46 (57.5)	16 (44.4)	62 (53.5)	
2	10 (12.5)	4 (11.1)	14 (12.1)	
3	9 (11.3)	1 (2.8)	10 (8.6)	
Decompressive procedure				0.55
No	58 (72.5)	28 (77.8)	86 (74.1)	
Yes	22 (27.5)	8 (22.2)	30 (25.9)	
ICP monitoring				0.39
No	31 (38.8)	17 (47.2)	48 (41.4)	
Yes	49 (61.3)	19 (52.8)	68 (58.6)	
**Imaging**				
Computed tomography (CT) imaging				0.56
0	0 (0.0)	0 (0.0)	0 (0.0)	
1-2	70 (87.5)	29 (80.6)	99 (85.3)	
3-4	8 (10.0)	6 (16.7)	14 (12.1)	
5+	2 (2.5)	1 (2.8)	3 (2.6)	
**Other**				
Ventilator days				
Median (IQR)	4.5 (4.0)	4.5 (7.0)	4.5 (6.0)	
Range	0-42	0-30	0-42	
Ventilator use				1.00
No	1 (1.3)	0 (0.0)	1 (0.9)	
Yes	79 (98.8)	36 (100)	115 (99.1)	
End-tidal capnography days				
Median (IQR)	1.0 (3.5)	0.0 (3.5)	1.0 (3.5)	
Range	0-7	0-7	0-7	
End-tidal capnography use				0.16
No	31 (38.8)	19 (52.8)	50 (43.1)	
Yes	49 (61.3)	17 (47.2)	66 (56.9)	
Central venous pressure				0.84
No	57 (71.3)	25 (69.4)	82 (70.7)	
Yes	23 (28.7)	11 (30.6)	34 (29.3)	
Arterial blood pressure				0.08
No	5 (6.3)	6 (16.7)	11 (9.5)	
Yes	75 (93.7)	30 (83.3)	105 (90.5)	

Unless otherwise indicated, values represent counts (n) and percentages. Within-category column totals may not sum to 100% due to missing data or rounding. Significance (sig.) represents p-value from chi-squared tests for categorical measures. Count variables report the total number of procedures or interventions performed during each patient’s PICU stay.

## Discussion

The COVID-19 pandemic posed challenges for the management of patients in hospitals across South America, including reduced availability of resources and longer response times [20–22]. Despite these stressors, this study observed little difference in hospital management and utilization of resources for treatment of severe TBI in South America before and during the COVID-19 pandemic. These findings suggest that hospitals were still able to prioritize the care of pediatric severe TBI patients, consistent with a recent PEGASUS study of pediatric severe TBI ICU care processes in South American hospitals between 2019 and 2021 that found that providers reported providing standard pediatric severe TBI care despite challenges presented by the COVID-19 pandemic [23].

In this study, vasopressor use and the number of surgical procedures decreased. Receipt of parenteral nutrition also dropped to zero during the pandemic period, though this finding was not statistically significant. Reasons for these observed variations in healthcare utilization merit discussion. First, children admitted prepandemic may have been more severely injured than during the pandemic, but neither GCS nor ISS were different between the two study periods. Second, types or patterns of injuries may have differed with fewer injuries requiring operative treatment during the pandemic than before. However, AIS scores did not differ between the two time periods. Third, patient outcomes were similar between the two time periods, suggesting that patients who were admitted during both time periods needed PICU care. It is possible that commonly used measures of injury severity did not fully capture injury severity as deemed by health services utilization. However, there may be a relationship between decreased utilization of some services (surgeries, arterial line placement, and vasopressor use) and select changes in clinical practices over time, such as the use of arterial lines even for those patients who had surgeries. Supply chain challenges during the pandemic may have resulted in reduced arterial line placement and parenteral nutrition. Finally, scheduled surgeries were often suspended during the COVID-19 pandemic, which may have freed resources for needed care for children with severe TBI, thereby explaining the mostly similar provision of prepandemic care in 2020. The changes in pediatric TBI care amidst the pandemic have been reported by providers of severe pediatric TBI care in South American sites prior to the RCT, in US emergency departments’ evaluation of TBI, observed rates of craniotomy in Africa, and through observations in US pediatric orthopedic trauma [23–26].

The overall mean hospital and PICU lengths of stay reported in this study were similar to the Colombian study by Arango and colleagues (28 and 13 days, respectively) [12]. Surgical procedures, such as decompressive procedures, were used more frequently in this study (26%) than either the previous Colombian study (9%) or Argentina study (12%), which may be due to changes in clinical practice over time. Additionally, in contrast to the Colombian study, severe TBI patients in this study had ICP monitoring more often (14% vs. 59%) [12]. In fact, the rate of ICP monitoring in this study was similar to previous research in Argentina (60%) [13] and remained consistent before and during the pandemic. Guidelines from the BTF suggest use of ICP monitoring for severely brain injured children, largely based on data from the US and Europe [10]. Despite observed differences in healthcare utilization in this study, patient care outcomes remained unchanged, suggesting that compensatory behaviors during the pandemic period did not adversely affect discharge disposition.

The results of this study should be interpreted in the context of its limitations. First, 72.4% of patients were transferred from another hospital, and the health resource utilization data for those hospitals is not available; however, patients were not ‘double-counted’. Second, while South American hospitals reportedly experienced resource shortages during the pandemic [20–22], this study focused on utilization of services for pediatric severe TBI and did not directly examine resource shortages in study hospitals. Given the substantial strain on healthcare systems during this period, we limited our data collection to a discrete set of measures in the first four months of the pandemic; nonetheless, this information provides perspective into hospital and PICU management of patients in the initial stages of the pandemic, during which severe restrictions were in place in the study countries, with COVID Stringency and Containment Indices of over 70% during the study time period [27]. Third, we did not power this study to address differences in location or cause of injury; future work could explore these the differences in these characteristics that were observed in this study. Finally, this was a secondary analysis of data from an RCT in South America. Hospital organizational characteristics were examined in the primary study, including staffing ratios of physicians and nurses to patients and resources to provide guideline-based care [18]. As expected and similar to the US, use of advanced neuromonitoring technologies were not available or used in the care of children with severe TBI. The generalizability of the findings from this study across different settings, including to other healthcare systems in South America, is uncertain. However, primary RCT data demonstrate that rates of guideline adherence at our study sites were comparable to or higher than those observed at U.S. institutions where this work was previously conducted.

Management of pediatric severe TBI is complex, and adherence to guidelines is associated with improvements in discharge outcomes, including discharge pediatric cerebral performance category scale and pediatric overall performance category scale in Argentina [13]. In resource-limited settings, however, adherence to pediatric severe TBI guidelines can be challenging due to lack of or limited staff, resources, and capacity [28].

Overall, this study shows that hospital disposition did not change during the pandemic period for children with TBI admitted to the PICU in South America, and supply chain shortages resulted in some compensatory care behaviors that did not affect most pediatric severe TBI care and outcomes at hospital discharge. Clinicians adapted to the pandemic challenges successfully.

## Supporting information

S1 File S1_Foreign_Language_Abstract.docx. (DOCX)
