# Supplementary material for: Pediatric severe TBI in South America: Healthcare resource utilization before and during the COVID-19 pandemic
Source: PLOS Glob Public Health. 2025 May 8;5(5):e0004318. doi: 10.1371/journal.pgph.0004318 (PMC12061101; doi:10.1371/journal.pgph.0004318)
Supplement: S1 File — (DOCX) [file pgph.0004318.s001.docx]

**RESUMEN**

**Antecedentes:** El traumatismo encefalocraneano (TEC) es una de las principales causas de morbilidad y mortalidad pediátrica en todo el mundo. El tratamiento de niños con TEC grave presenta desafíos particulares en entornos con recursos limitados. Comprender el uso de los servicios de atención médica durante la hospitalización por TEC grave en diversos entornos es crucial para informar mejoras en la práctica clínica, los resultados de los pacientes y para reducir la carga del TEC. El propósito de este estudio fue examinar el uso de servicios hospitalarios en niños con TEC grave en el contexto sudamericano. **Métodos:** Este es un análisis secundario de datos recopilados durante el período inicial de un ensayo clínico aleatorizado de guías de manejo clínico del TEC grave en América del Sur. Se identificaron pacientes pediátricos (<18 años) con TEC grave en 16 hospitales en Argentina, Chile y Paraguay entre el 1 de septiembre de 2019 y el 13 de julio de 2020. Se recopilaron datos demográficos, características de la lesión, presentación clínica, uso hospitalario, intervenciones quirúrgicas e imágenes. Se calcularon estadísticas descriptivas para todas las medidas. Se examinaron las diferencias del uso en dos períodos, prepandémico (1 de septiembre de 2019-10 de marzo de 2020) y durante la pandemia (11 de marzo-13 de julio de 2020), utilizando pruebas t de Student y pruebas de chi-cuadrado. **Resultados:** Un total de 116 pacientes (edad promedio: 7.0 años) fueron ingresados con TEC grave durante el período del estudio (n=80 prepandémico y 36 durante la pandemia). No hubo diferencias en datos demográficos, en mecanismos de lesión o de resultados al alta entre los dos períodos. El uso del monitoreo de la presión intracraneal (PIC) se mantuvo por encima del 50% en ambos períodos. El uso de vasopresores disminuyó del 63.8% antes de la pandemia al 33.3% durante la misma (p<0.01) y también disminuyó el número de cirugías (p=0.05). **Conclusiones:** El déficit en la cadena de suministros dio lugar a comportamientos compensatorios del cuidado agudo que no afectaron en gran medida la atención y los resultados del TEC pediátrico más grave al momento del alta hospitalaria. Los profesionales de la salud se adaptaron con éxito a los desafíos de la pandemia.
